# Thyroid Nodules Located in the Lower Pole Have a Higher Risk of Malignancy than Located in the Isthmus: A Single-Center Experience

**DOI:** 10.1155/2021/9940995

**Published:** 2021-07-14

**Authors:** Gulhan Duman, Baris Sariakcali

**Affiliations:** Division of Endocrinology and Metabolism, Department of Internal Medicine, Faculty of Medicine, Sivas Cumhuriyet University, Sivas 58140, Turkey

## Abstract

**Purpose:**

The aim of our study is to investigate whether thyroid nodules (TNs) localization has value as a predictor of malignancy. Ultrasonography provides very valuable information in the evaluation of TNs, but it does not correlate perfectly with histopathologic findings. Therefore, studies that will include new diagnostic methods that can improve these unknowns can be welcomed gratefully.

**Methods:**

This study was carried out retrospectively in a tertiary care center from September 2016 to January 2020. The study included 862 adult patients who have one or more nodules. Ultrasonography of characteristics of nodules such as echogenicity, content, margins, calcifications, size, and localization was recorded. Fine-needle aspiration biopsy (FNAB) was performed on dominant and suspicious 1142 nodules.

**Results:**

The patients were composed of 692 (80.3%) females and 170 (19.7%) males. Compared to nodules located in the isthmus; the malignancy risk increased 8.39 (OR: 8.39 (2.34–30.12), *p* = 0.001) times in the lower pole, 4.27 (OR: 4.27 (1.16–15.72), *p* = 0.029), times in the middle pole, 8.09 (OR: 8.09 (2.11–30.94), *p* = 0.002) times in the upper pole, and 7.63 (OR: 7.63 (1.95–29.81), *p* = 0.003) times in the nodules covering the whole of the lobe. Although the most nodular location was in the middle pole, the risk of malignancy was less than that in the lower and upper poles.

**Conclusions:**

Unlike the other localization studies, we found a higher risk of malignancy in the lower and similarly upper thyroid poles. Besides well-defined malignancy indicators in the literature and guidelines, localization information is promising for this purpose in the future.

## 1. Introduction

Thyroid nodules (TNs) have been observed as extremely prevalent and are commonly described in patients with no symptoms who are going through assessment for different medical conditions. Their frequency could be affected by many agents, such as iodine sufficiency status and age, and detection rates vary in accordance with the modality of imaging used and the experience of the operator [1, 2]. In high-resolution ultrasonography (US), the prevalence of TNs lines up from 19% to 68%, with a higher rate in older adults and women. Besides, increased utilization of imaging modalities such as computed tomography (CT) scan, sonographic carotid evaluation, magnetic resonance imaging (MRI), and 1% to 2% with 18-fluorodeoxyglucose positron emission tomography (PET) may have contributed to increased detection of TNs [3–5].

Thyroid cancer is thought to be the most prevalent endocrine cancer, accounting for 2.1% of all cancers worldwide [6]. The rising case of thyroid cancer worldwide is linked to the wider use of diagnostic techniques such as ultrasound scans coupled with fine-needle aspiration biopsy (FNAB) of incidentally found small nodules [7, 8]. According to the Surveillance, Epidemiology, and End Results Program (SEER), the incidence has increased annually since the 1970s, the number of deaths due to thyroid cancer has remained relatively constant [9]. Overtreatment and overdiagnosis of small papillary thyroid cancers have culminated in a reevaluation of the management approach for the tumors, within a debate on when and how to treat these sort of small, often serendipitously discovered lesions [10, 11].

The fact that high risk features for thyroid cancer and nodule measure on a US scan can help indicate the requirement for further diagnostic survey within FNAB [12, 13]. The well-known malign features of nodules on US have marked hypoechogenicity compared to background thyroid, microcalcifications, and irregular margins [12, 14].

In daily practice, there are a few guides that will make it easier for physicians to make a follow-up or biopsy decision about the nodule. These rules have been created for permitting US imaging to be utilized for the recognizable proof and stratification of nodules which are based on the chance of malignancy. The foremost known one involves the American Thyroid Association (ATA), the American College of Radiology's Thyroid Imaging Reporting and Data System (TIRADS), the European Thyroid Association (EU-TIRADS), and the Korean Society of Thyroid Radiology (K-TIRADS), which have been developed and validated which have been based on existing multi-institutional information and master supposition [3, 12, 13, 15].

Although the US provides many highly valuable clinical insights about the thyroid gland, its diseases, and adjacent structures, the results of the thyroid US do not correlate perfectly with histopathologic findings. Therefore, studies that will include a new diagnostic method that can improve these unknowns can be welcomed gratefully.

There are a few publications suggesting that TNs localization may also be a factor in the development of thyroid cancer recently. These studies are not numerous and the results are inconsistent [16, 17]. The main objective of this research is to investigate if there has been a correlation between the localization of thyroid nodules and thyroid cancer.

## 2. Materials and Methods

The research protocol was affirmed by the Human Research Ethics Committee of Sivas Cumhuriyet University (2020-10/11). Patients who were admitted to our outpatient clinic between September 2016 and March 2020 and performed FNAB were included in the study. During this period, 1142 FNAB was performed on 862 adult patients who have one or more nodules. Written consent has been obtained from each patient or subject after a full explanation of the purpose and nature of all procedures used. All of the final follow-up ultrasound examinations in the study were performed by two experienced endocrinologists.

Fine-needle aspiration biopsy was performed by determining dominant and suspicious nodules. Information such as the gender and age of the patient as of the US-FNAB, in addition to cytology and histopathology results, were obtained from the hospital's electronic medical case records and case notes in our clinic.

### 2.1. Ultrasonography

Thyroid US was performed with a HITACHI EUP-L74 M model ultrasound linear array 7.5 MHz probe. We recorded the following ultrasound features of nodules: echogenicity (iso/hyperechoic, hypoechoic, and very hypoechoic), content, taller-than-wide irregular-margins, presence of microcalcifications (<1 mm), macrocalcifications (>1 mm), disrupted rim calcifications, colour Doppler vascular activity, and the composition of the nodule. A taller-than-wide shape was classified as one involving anteroposterior to transverse diameter ratio ≥1 [18, 19].

Colour Doppler vascularity grades are defined as Type I, a total absence of flow signal in the nodule; Type II, exclusive perinodular flow signals; and Type III, intranodular flow with numerous vascular poles chaotically organized, including or regardless of critical perinodular vessels [20]. We graded Doppler vascularity as no vascular activity (Type 0), perinodular vascular activity (Type I), intranodular vascular activity (Type II), and intranodular and perinodular (chaotic) vascular activity (Type III).

In addition, nodule size measured at the maximum dimension and localization were evaluated. The thyroid left and right lobes was subjectively divided into 3 pole vertically; the upper, middle, and lower poles. If the nodule is not definitely located in the upper, lower or middle pole and shows extension to the other pole, the location of 2/3 of the nodule was recorded as that pole.

### 2.2. Pathological Evaluation

US-guided FNAB was conducted according to relevant guidelines and regulations. Fine needle aspiration biopsy was performed for all sort of nodules which were greater than 1 cm, and in one of the presence of suspicious US findings such as hypoechogenicity, microcalcification, border irregularity, taller than wide shape, and interrupted rim calcifications, biopsy was also performed on nodules >1 cm. In patients, they have multiple nodules (MTN), and the diagnostic approach was similar to that with a solitary nodule (STN). The condition of being euthroid was not sought in patients. However, thyroid scintigraphy with technetium was performed first in patients with thyroid stimulating hormone (TSH) levels lower than the reference range or low-normal. No biopsy was taken from nodules that were hyperactive on scintigraphy.

Cytological evaluation of FNAB specimens was made by the recommendation of the Bethesda System for Reporting Thyroid Cytopathology (BSRTC) in 2008 [21]. Based on the cytological results, which were categorized into six groups, the findings are nondiagnostic (ND) (Bethesda 1), benign (Bethesda 2), atypical of undetermined significance/follicular lesions of undetermined significance (AUS/FLUS) (Bethesda 3), follicular neoplasm/suspicious for follicular neoplasm (FN/sFN) (Bethesda 4), suspicious for malignancy (sM) (Bethesda 5), and malignant (Bethesda 6) [22].

Nodules with nondiagnostic or indeterminate (Bethesda categories 1, 3, and 4) were excluded unless precise FNAB results or after resection the histologic results were available. Nodules with suspicious malignancy FNA results (Bethesda category 5) were also excluded unless there was a subsequent definitive surgery to confirm the diagnosis. Those diagnosed as benign (Bethesda 2) or malignant (Bethesda 6) in FNAB were accepted definitive diagnoses. Almost all malignant and suspicious malignancy nodules were surgically removed, while most benign (Bethesda 2) nodules were not. Bethesda category 5 was evaluated in the malignant group because it estimated %100 malignant postoperatively.

### 2.3. Statistical Analysis

The compliance of the data to normal distribution was evaluated by the histogram, q-q graphs, and Shapiro–Wilk test. Variance homogeneity was tested with the Levene test. Independent two-sample *t*-test and Mann–Whitney *U* test were used for quantitative variables in comparisons between paired groups. One-way analysis of variance (ANOVA) and Kruskal–Wallis test were used in comparisons of more than two groups. Tukey and Bonferroni tests were applied for multiple comparisons. Chi-square analysis was used to compare categorical variables. Logistic regression analysis was performed to determine the risk factors affecting malignancy status for both FNAB and postoperative pathology. As a result of logistic regression analysis, multiple logistic regression analysis was performed with variables with *p* < 0.200. The backward conditional method was used in multiple logistic regression analyses. The analysis of the data was carried out in IBM SPSS 22 statistics software. The significance level was accepted as *p* < 0.05.

## 3. Results

The study was completed with 862 patients and 1142 nodules undergone FNAB. Patients' age, gender, nodule diameter, TSH, Hashimoto's, and hyperthyroidism values are given in Table 1.

The ecogenic dispertion of nodules were 520 (49.1%) isoechoic, 428 (40.4%) hypoechoic, 41 (3.9%) hyperechoic, and 71 (6.7%) mixed echoic. 278 of the nodules (43.2%) had cystic areas, 42 (6.5%) necrosis, 223 (34.6%) microcalcification, 28 (4.3%) macrocalcification, 27 (4.2%) border irregularity, 23 (3.6%) intermittent wall calcification, and 23 (3.6%) taller than wide ultrasonographic features. The patients Doppler US results were 148 (14.7%) no blood flow, 507 (50.3%) peripheral, 80 (7.9%) central, and 272 (27.0%) central and peripheral blood flow.

The location distribution of nodules on the left lobe is 137 (27.9%) in the lower pole, 175 (35.6%) in the middle, 93 (18.9%) in the upper pole, and 86 (17.5%) of the whole lobe. The localization of the nodules on the right lobes is 136 (28.6%) in the lower, 189 (39.7%) in the middle, 71 (14.9%) in the upper, and 80 (16.8%) cover near the whole lobe. When the sum of the upper, middle, and lower pole nodules of the left and right lobes was evaluated, the localization distribution was 273 (28.2%) in the lower, 364 (37.6%) in the middle, 164 (17.0%) in the upper pole, and 166 (17.2%) covered almost the entire lobe. The distribution according to isthmus is 53 (34.4%) in the left lobe junction, 33 (21.4%) in the central, and 68 (44.2%) in the right lobe junction.

Comparisons in FNAB results were similar in benign and malignant patient groups, and no statistically significant difference was found (*p* > 0.05). Nodule number was higher in the benign group than the malignant group (*p* = 0.004), and MTN rate higher in the benign group and non-MTN in the malignant group (*p*= 0.008) (Table 2).

In comparison to benign and malignant cytology on FNAB, a statistically significant relationship was found between echogenicity, US findings, and Doppler blood flow (*p* < 0.001). While isoechogenicity, peripheral Doppler flow, and cystic content were significantly higher in benign nodules (*p* < 0.001), hypoechogenicity border irregularity, and absence of Doppler flow or central flow were significantly higher in malignant nodules (*p* < 0.001) (Table 3).

As a result of FNAB, there was no statistically significant difference between these groups with the location of the right lobe, left lobe, and in total right-left lobes and their upper, middle, and lower poles (*p* > 0.05).

The patient groups diagnosed as benign, malignant, and incidental tumor in postoperative pathology were compared according to some characteristics shown in Table 4. In the incidental group nodule size, nodule number and the rate of MTN is higher, but the TSH levels are lower than the other groups (*p* = 0.013, *p* = 0.020, and *p* = 0.009, respectively). TSH levels higher in the malignant group and 10–20 mm nodule size more both in benign and malignant groups (*p* = 0.001 and *p* = 0.022, respectively).

Ecogenicity, US features, and Doppler US findings were compared postoperatively (Table 5). Nodules with isoechoic, cystic content, and peripheral Doppler blood flow are significantly more in the benign group. Nodules with hypoechoic, necrosis, border irregularity, and taller than wide shape, central, and central plus peripheral blood supply are more in the malignant group (*p* = 0.002, *p* < 0.001, and *p* < 0.001, respectively). Mixed echoic and central blood flow nodules are markedly higher in the incidental group (*p* = 0.002 and *p* < 0.001, respectively**)**.

The postoperative pathological groups of benign, malignant, and incidental groups were compared with thyroid tissue localization and cancer relation (Table 6). There was no significant relation with left lobe localization and these groups (*p* > 0.05), but nodules covering all lobe in the right lobe is more in the incidental group (*p* = 0.018). In the sum of right and left lobes, isthmus and middle pole localization is high in the benign group, and whole lobe location is high in the incidental group (*p* = 0.001).

According to FNAB, results were examined in order to evaluate the risk factors affecting malignancy (Table 7). Age, gender, TSH levels, Hashimoto's, nodule size, and nodule location did not constitute a statistically significant risk factor for malignancy (*p* > 0.05). In univariate analysis, MTN was related to a 65.7% lower rate risk of malignancy (OR: 0.34, *p* = 0.011). The number of nodules found to be a risk factor for malignancy (*p* = 0.024), and one-unit increase in the number of nodules reduces the risk of malignancy by 30% (OR: 0.70). The risk of malignancy is %89.1 less in patients with peripheral blood supply than in those without blood supply nodules in Doppler US (OR: 0.10).

In multivariate analysis, MTN variables were insignificant (*p* > 0.05), but the number of nodules and Doppler variables was found to be significant independent risk factors (*p* < 0.05). One-unit increase in the number of nodules reduces the risk of malignancy by 30% (OR: 0.70, *p* = 0.034). According to the Doppler result, the risk of malignancy is %90.3 less in those with peripheral blood supply than those without blood supply (OR: 0.09, *p* = 0.001).

Postoperative pathological results were evaluated to determine the risk of malignancy (Table 8) Variables of gender, TSH levels, Hashimoto's, MTN, nodule size, and nodule number were not a statistically significant risk factor for malignancy (*p* > 0.05).

In univariate analysis, one-unit increase in age decreases malignancy by %2.2 (OR: 0.97, *p* = 0.007) and hypoechoic nodules increase the malignancy risk 1.74 times compared to isoechoic ones (OR: 1.74). In US findings compared to cystic areas, those with necrosis were 6.46 (OR: 6.46, *p* < 0.001), those with microcalcification were 2.78 (OR: 2.78, *p* = 0.002), those with border irregularities were 13.44 (OR: 13.44, *p* < 0.001), and those taller than wide were 38.40 (OR:38.40, *p* < 0.001) times increased risk of malignancy. In Doppler US, those with central blood supply increase the risk of malignancy by 3.55 (OR: 3.55 (1.65–7.62)) times compared to those without blood supply.

The location information variable has a statistically significant effect on malignancy status (*p* < 0.05), compared to those whose nodules in the isthmus: lower location nodules 4.45 (OR: 4.45 (1.70–11.62), *p* = 0.002), upper ones 4.57 (OR: 4.57 (1.67–12.46), *p* = 0.003), the whole of the lobe 3.17 (OR: 3.17 (1.12–8.96), *p* = 0.029) times riskier for malignity.

Ecogenicity variable was insignificant in multivariate analysis (*p* > 0.05), while age, US findings, Doppler, and the sum of the left right lobe location variables were found to be significant independent risk factors (*p* < 0.05). A one-unit increase in age reduces the risk of malignancy by %2.3 (OR: 0.97, *p* = 0.035). According to US findings compared to cystic areas, those with necrosis 7.09 (OR: 7.09, *p* < 0.001), those with microcalcifications 3.13 (OR: 3.13, *p* = 0.002), those with border irregularity 9.82 (OR: 9.82, *p* < 0.001), taller than wide 50.05 ((OR: 50.05), *p* < 0.001)) times increased risk of malignancy. Those with central blood supply increase the risk of malignancy by 2.88 (OR: 2.88 (1.10–7.50), *p* = 0.030) times compared to those without blood supply.

The location variable has a statistically significant effect on malignancy as an independent risk factor (*p* < 0.05) by multivariate analysis, according to nodules located in isthmus; there is 8.396 (OR: 8.39 (2.34–30.12), *p* = 0.001) fold increase in malignancy risk in the lower pole, 4.27 (OR: 4.27 (1.16–15.72), *p* = 0.029), those in the middle pole, 8.09 (OR: 8.09 (2.11–30.94), *p* = 0.002) in the upper pole, and a 7.63 (OR: 7.63 (1.95–29.81), *p* = 0.003) fold increase in the nodules covering the whole of the lobe.

## 4. Discussion

In this retrospective single-center study, we aimed to investigate the relationship of nodule localization with malignancy.

Thyroid nodules are fourfold more frequent in women when compared to men. In literature context in male sex and age lower than 20 and over 60 years are detailed to be related within a higher thyroid malignancy risk [23]. Thyroid cancer had a 2.9 times higher rate in women (female: male proportion 16.3 : 5.7) [24]. We found a similar rate of thyroid cancer in females and males in both FNAB and postoperative pathologic results.

There are conflicting results as to whether nodule size may be used to distinguish at malignity risk for lesions. Zhang et al. showed that factors such as age, gender (male more than females), radiation exposure, and family history are associated with thyroid cancer, but not with thyroid nodule size [25]. Most of our benign and malignant nodules were 10–20 mm (*p* = 0.022), but the nodule size of the patient with incidental tumors was larger than these two groups (*p* = 0.013). As in the study of Popowicz et al., the risk of malignancy was higher in nodules with hypoechoic, microcalcification, and taller than wide features [26].

However, logistic regression analysis showed echogenicity variable was significant in univariate but insignificant in multivariate analyzes (*p* > 0.05), while age, US findings, Doppler, and the sum of left right lobe location variables were found to be significant independent risk factors in malignity (*p* < 0.05). An increase in age reduces the risk of malignancy by %2.3 (OR: 0.97). According to US findings compared to cystic areas; those with necrosis 7.09 (OR: 7.09)), those with microcalcifications 3.13 (OR: 3.13), those with border irregularity 9.82 (OR: 9.82), taller than wide 50.05 (OR: 50.05) times increase the risk of malignancy.

It is not surprising that our findings were consistent with ultrasonographic features, echogenicity, and Doppler US, which are the main determinants of malignancy in guidelines and literature.

Gul et al. [27] did not detect a cytologically significant difference. In the postoperative results, patients with STN were found to be significantly associated with malignancy than those with MTN. Kumar et al. [28] revealed that the chance of thyroid cancer was lower in patients within MTN than in the ones with STN. In a meta-analysis in 12 studies, SN was associated with malignancy [29]. According to these studies, we found the number of nodules to be a risk factor for malignancy (*p* = 0.024), and one-unit increase in the number of nodules reduces the risk of malignancy by 30% (OR: 0.70) and MTN was related to 65.7% lower rate risk of malignancy (OR: 0.34, *p* = 0.011) in our study.

In a retrospective survey of 1,083 TNs, Moon et al. revealed that intranodular vascularity was detected in 31% of benign TNs in comparison with 17% of malignant nodules [30]. However, in the meta-analysis covered 41 surveys, 15 studies discovered that intranodular vascularization was associated with a higher chance of malignancy [29]. Several studies have suggested that intranodular vascularity might have a superior relation with malignancy in follicular thyroid cancers than papillary thyroid cancers [31].

When compared to those without blood supply in Doppler US postoperative results, those with central blood supply increase the risk of malignancy OR, 2.88 times, while the risk of malignancy is %90.3 less in those with peripheral blood supply (OR:0.09, *p* = 0.001) in our FNAB results. Therefore, we think that the combination of several suspicious US highlights into sonographic Doppler features develops the expectation of malignancy chance in these nodules.

Ramundo et al. evaluated 832 TNs which experienced US-guided FNAB, 557 of them had a definitive final diagnosis (513 benign, 44 malignant). As a result, they found that thyroid malignancy was more frequent in the middle lobe (OR, 9.74). Multivariate analysis revealed a high OR for malignancy (was affirmed for nodules with a) in the mid-lobar location (OR, 7.70). The upper pole location also indicated a substantial relation with malignancy (OR, 6.92) [16].

Zhang et al. [25], in their small sample size study with 219 thyroid nodules, reviewed the data retrospectively. Most nodules (79.3%) were placed within the lower pole, while 9.6% were found in the upper pole and 6.9% in the middle pole. A crucially higher frequency of malignancy was monitored in the upper pole (22.2%) in comparison with the lower pole (4.7%) and middle pole (15.4%). The odds of malignancy in the upper pole were 4 times higher than in other areas (OR, 4.6). Some authors associate this high odds ratio level with being a small-scale study group, as we thought.

Amisten and Betts found a higher frequency of malignancy among nodules located in the upper pole of the gland (22.2%) compared to the lower (4.7%) and middle (14.3%) poles, malignancy in upper pole nodules four times higher than nodules in other locations [32]. Sibai and Zahedi found that of the 50 malignant nodules, 34 were located in the upper lobe (68%) and 16 were located in the lower lobe (32%). The odds of malignant nodules being located in the upper lobe were 9.6 times more than the odds in the lower lobe (*p* < 0.001) [33].

Based on a large, retrospective, multicentric survey in 9,535 samples, a malignant diagnosis was more than twice as common in the isthmus compared with the lateral lobes (8.1% vs. 3.6% and 3.1%, respectively, for the right and left lobes (*p* < 0.001 for both) and 8.1% vs. 3.4% for the 2 lobes together (*p* < 0.001)) [34].

In a new study in which 3313 adult patients with six centers, it was found that malignant TNs were significantly higher in the isthmus. It is followed by upper TNs, and then middle TNs compared to lower TNs. [17].

Contrary to these latest study according to nodules located in isthmus, we found 8.39 (OR: 8.39 (2.34-30.12), *p* = 0.001) fold increase in malignancy risk in the lower pole, 8.09 (OR: 8.09 (2.11-30.94), *p* = 0.002) in the upper pole, and 4.27 (OR: 4.27 (1.16-15.72), *p* = 0.029) in the middle pole. The nodules are covering the whole of the lobe, mostly 7.63 (OR: 7.63 (1.95–29.81), *p* = 0.003) fold increase risk of malignancy, especially in incidental tumors.

As a suggested mechanism of the nodules in the thyroid upper pole to malignant transformation is the increase of reactive oxygen radicals caused by the lack of direct drainage of the tortuos veins and their triggering malignant mutations in the nodule. In addition, the diagnostic radiological procedures used during dental procedures are blamed, but the reason for the increase in malignancy of the nodules in the isthmus and middle pole is unknown [16, 17, 25, 32–34]. We speculate that the more common and uncontrolled use of computerized tomography for diagnostic purposes in our country may be related to our lower pole result.

## 5. Conclusion

As a result, there are various outcomes regarding nodule localization and malignancy in the upper pole, middle pole, isthmus, and finally, the lower and upper poles shown in the last study. Geographic regions, nutrition, habits, and environmental and epigenetic factors may also play a role in the relationship between different results of localization and malignancy. Therefore, perhaps we should be more cautious in interpreting these results and conduct larger-scale controlled studies before recommending the relationship of localization with malignancy to guides.

## Figures and Tables

**Table 1:** Demographic and clinical characteristics.

Variables	Descriptive statistics (nodules *n* = 1142)
*Age*	52.29 ± 12.86

*Gender*	
Female	692 (80.3)
Male	170 (19.7)

*TSH (mIU/L)*	1.35 (0.75–2.43)

*Nodule size (mm)*	17.80 (12.90–25.60)
<10	84 (7.4)
10–20	579 (50.8)
20–40	395 (34.6)
>40	82 (7.2)

*Nodule number*	2.0 (1.0–4.0)

*Multinodularity (MTN)*	
No	380 (33.4)
Yes	758 (66.6)

*Hashimoto's*	
No	1017 (89.8)
Yes	116 (10.2)

*Hyperthyroidism*	
No	1072 (94.7)
TMNG	39 (3.4)
Graves	8 (0.7)
Iatrogenic	13 (1.1)

Data are expressed in mean ± standard deviation (%) and median (1st quartile-3 rd quartile). mIU/L: milli-international units per liter; mm: millimeter; TMNG: toxic multinodulary goiter.

**Table 2:** Demographic and clinical features of benign and malignancy in fine-needle aspiration biopsy (FNAB) result.

Variables	FNAB		*p*
	Benign (*n* = 679)	Malignancy (*n* = 24)	
*Age*	52.44 ± 12.82	50.38 ± 14.78	0.441

*Gender*			
Female	545 (80.30)	19 (79.20)	0.799
Male	134 (19.70)	5 (20.80)	
*TSH (mIU/L)*	1.32 (0.74–2.40)	2.00 (0.96–2.96)	0.115

*Nodule size (mm)*	18.20 (13.15–26.00)	15.25 (10.55–23.75)	0.088
<10	41 (6.10)	4 (16.70)	0.156
10–20	339 (50.10)	11 (45.80)	
20–40	255 (37.70)	7 (29.20)	
>40	42 (6.20)	2 (8.30)	

*Nodule number (n)*	2.00 (1.00–4.00)	1.00 (1.00–2.00)	**0.004**

*MTN*			
No	195 (28.80)^a^	13 (54.20)^b^	**0.008**
Yes	481 (71.20)^a^	11 (45.80)^b^	

*Hashimoto's*			
No	610 (90.20)	21 (87.50)	0.723
Yes	66 (9.80)	3 (12.50)	

*Hyperthyroidism*			
No	641 (95.00)	24 (100.00)	0.999
TMNG	20 (3.00)	0 (0.00)	
Graves	6 (0.90)	0 (0.00)	
Iatrogenic	8 (1.20)	0 (0.00)	

Data are expressed mean ± standard deviation *n* (%) and median (1st-3 rd. quartile). Similar letters on the same line indicate similarity between groups and different letters indicate differences between groups. TSH: thyroid stimulatingt hormone, mIU/L: milli-international units per liter; mm: millimeter; MTN: multinodularity, TMNG: toxic multinodulary goiter. *Note*. Bethesda category 5, because it estimated %100 malignant postoperatively, and it was evaluated in the malignant group.

**Table 3:** Evaluation of FNAB results according to ultrasonographic features.

Variables	FNAB		*p*
	Benign (*n* = 633)	Malignant (*n* = 24)	
*Ecogenicity*			
Isoechoic	324 (51.2)^a^	1 (4.2)^b^	**<0.001**
Hypoechoic	226 (35.7)^a^	22 (91.7)^b^	
Hyperechoic	31 (4.9)^a^	0 (0.0)^a^	
Mixed echoic	52 (8.2)^a^	1 (4.2)^a^	

*US findings*			
Cystic areas	175 (49.9)^a^	1 (4.2)^b^	**<0.001**
Necrosis	21 (6.0)^a^	2 (8.3)^a^	
Microcalcification	117 (33.3)^a^	10 (41.7)^a^	
Macrocalcification	14 (4.0)^a^	0 (0.0)^a^	
Border irregularity	8 (2.3)^a^	8 (33.3)^b^	
Intermittent wall calcification	10 (2.8)^a^	0 (0.0)^a^	
Taller than wide	6 (1.7)^a^	3 (12.5)^b^	

*Doppler US*			
No blood flow	71 (12.0)^a^	6 (26.1)^b^	**<0.001**
Peripheral blood flow	325 (54.7)^a^	3 (13.0)^b^	
Central blood flow	40 (6.7)^a^	8 (34.8)^b^	
Central and peripheral blood flow	158 (26.6)^a^	6 (26.1)^a^	

Data expressed as *n* (%). Similar letters on the same line indicate similarity between groups and different letters indicate differences between groups. US: ultrasonography.

**Table 4:** Comparison of postoperative pathological outcomes with demographic and clinical characteristics.

Variables	Postoperative pathological outcomes			*p*
	Benign (*n* = 998)	Malignant (*n* = 102)	Incidental (*n* = 42)	
*Age*	52.57 ± 12.92^a^	48.93 ± 12.50^b^	53.62 ± 11.19^ab^	**0.019**

*Gender*				
Female	798 (80.0)	82 (80.4)	30 (71.4)	0.391
Male	199 (20.0)	20 (19.6)	12 (28.6)	

*TSH (mIU/L)*	1.33 (0.74–2.40)^a^	1.88 (0.97–2.70)^b^	0.97 (0.31–1.79)^c^	**0.001**

*Nodule size (mm)*	17.6 (13.0–25.0)^a^	16.0 (12.0–27.5)^a^	24.1 (18.0–33.5)^b^	**0.013**
<10	69 (6.9)^a^	11 (10.8)^a^	4 (9.5)^a^	**0.022**
10–20	519 (52.1)^a^	49 (48.0)^a^	11 (26.2)^b^	
20–40	340 (34.1)^a^	34 (33.3)^a^	21 (50.0)^a^	
>40	68 (6.8)^a^	8 (7.8)^a^	6 (14.3)^a^	

*MTN*				
No	338 (34.0)^a^	37 (36.6)^a^	5 (11.9)^b^	**0.009**
Yes	657 (66.0)^a^	64 (63.4)^a^	37 (88.1)^b^	

*Nodule number*	2.00 (1.00–4.00)^a^	2.00 (1.00–3.00)^a^	3.00 (2.00–5.25)^b^	**0.020**

*Hashimoto's*				
No	895 (89.9)	91 (90.1)	31 (83.8)	0.471
Yes	100 (10.1)	10 (9.9)	6 (16.2)	

*Hyperthyroidism*				
No	941 (94.6)^ab^	100 (99.0)^a^	31 (86.1)^b^	**0.009**
TMNG	37 (3.7)^a^	1 (1.0)^a^	1 (2.8)^a^	
Grave's	8 (0.8)^a^	0 (0.0)^a^	0 (0.0)^a^	
Iatrogenic	9 (0.9)^a^	0 (0.0)^a^	4 (11.1)^b^	

Data are expressed in mean ± standard deviation *n* (%) and median (1st quartile-3 rd. quartile). Similar letters on the same line indicate similarity between groups and different letters indicate differences between groups.

**Table 5:** Comparison of postoperative pathological results with US findings.

Variables	Postoperative pathological results			*p*
	Benign (*n* = 925)	Malignant (*n* = 100)	Incidental (*n* = 35)	
*Ecogenicity*				
Isoechoic	471 (50.9)^a^	39 (39.0)^ab^	10 (28.6)^b^	**0.002**
Hypoechoic	360 (38.9)^a^	52 (52.0)^b^	16 (45.7)^ab^	
Hyperechoic	37 (4.0)^a^	3 (3.0)^a^	1 (2.9)^a^	
Mixed echoic	57 (6.2)^a^	6 (6.0)^a^	8 (22.9)^b^	

*US findings*				
Cystic areas	252 (48.1)^a^	15 (17.4)^b^	11 (32.4)^ab^	**<0.001**
Necrosis	26 (5.0)^a^	10 (11.6)^b^	6 (17.6)^b^	
Microcalcification	181 (34.5)^a^	30 (34.9)^a^	12 (35.3)^a^	
Macrocalcification	24 (4.6)^a^	2 (2.3)^a^	2 (5.9)^a^	
Border irregularity	15 (2.9)^a^	12 (14.0)^b^	0 (0.0)^ab^	
İntermittent wall calcification	19 (3.6)^a^	1 (1.2)^a^	3 (8.8)^a^	
Taller than wide	7 (1.3)^a^	16 (18.6)^b^	0 (0.0)^a^	

*Doppler US*				
No blood flow	131 (15.0)^a^	14 (14.4)^a^	3 (8.6)^a^	**<0.001**
Peripheral blood flow	469 (53.6)^a^	26 (26.8)^b^	12 (34.3)^ab^	
Central blood flow	50 (5.7)^a^	19 (19.6)^b^	11 (31.4)^b^	
Central and peripheral blood flow	225 (25.7)^a^	38 (39.2)^b^	9 (25.7)^ab^	

Data are expressed as *n* (%). Similar letters on the same line indicate similarity between groups and different letters indicate differences between groups.

**Table 6:** Nodule localization relationship with postoperatively pathologic groups.

Variables	Postoperatively pathologic groups			*p*
	Benign (*n* = 679)	Malignant (*n* = 101)	Incidental (*n* = 42)	
*Left lobe*				
Lower	114 (26.8)	19 (41.3)	4 (20.0)	0.309
Middle	158 (37.2)	10 (21.7)	7 (35.0)	
Upper	79 (18.6)	9 (19.6)	5 (25.0)	
Whole lobe	74 (17.4)	8 (17.4)	4 (20.0)	

*Right lobe*				
Lower	118 (28.8)^a^	16 (32.0)^a^	2 (12.5)^a^	**0.018**
Middle	170 (41.5)^a^	15 (30.0)^a^	4 (25.0)^a^	
Upper	57 (13.9)^a^	12 (24.0)^a^	2 (12.5)^a^	
Whole lobe	65 (15.9)^a^	7 (14.0)^a^	8 (50.0)^b^	

*Sum of right and left lobes*				
Isthmus	147 (15.0)^a^	5 (5.0)^b^	2 (5.3)^ab^	
Lower	231 (23.6)^a^	35 (34.7)^b^	6 (15.8)^ab^	**0.001**
Middle	327 (33.4)^a^	25 (24.8)^a^	11 (28.9)^a^	
Upper	135 (13.8)^a^	21 (20.8)^a^	7 (18.4)^a^	
Whole lobe	139 (14.2)^a^	15 (14.9)^ab^	12 (31.6)^b^	

Data are expressed as *n* (%). Similar letters on the same line indicate similarity between groups and different letters indicate differences between groups.

**Table 7:** Evaluation of risk factors affecting malignancy status for FNAB results.

Variables	Univariate		Multivariate	
	OR (%95 CI)	*p*	OR (%95 CI)	*p*
*Age*	0.99 (0.95–1.01)	0.44		

*Gender*				
Female	1	—		
Male	1.07 (0.39–2.92)	0.89		

*TSH*	1.14 (0.89–1.45)	0.28		
*Hashimoto's*	1.32 (0.38–4.55)	0.65		

*MTN*				
No	1	—		
Yes	0.34 (0.15–0.77)	**0.011**		

*Nodule size*	0.96 (0.92–1.01)	0.174		
*Nodule number*	0.70 (0.51–0.95)	**0.024**	0.70 (0.50–0.97)	**0.034**

*Doppler US*				
No blood flow	1	—	1	—
Peripheral blood flow	0.10 (0.02–0.44)	**0.002**	0.09 (0.02–0.40)	**0.001**
Central blood flow	2.36 (0.76–7.30)	0.134	2.16 (0.64–7.25)	0.211
Central and peripheral blood flow	0.44 (0.14–1.44)	0.179	0.38 (0.11–1.26)	0.115

*Sum of right and left lobes*				
Isthmus	1	—		
Lower	3.38 (0.39–29.44)	0.26		
Middle	4.76 (0.60–37.72)	0.13		
Upper	5.77 (0.68–48.78)	0.10		
Whole lobe	2.14 (0.19–24.09)	0.53		

CI: confidence interval; OR: odds ratio.

**Table 8:** Evaluation of risk factors affecting malignancy according to postoperative pathological results.

Variables	Univariate		Multivariate	
	OR (%95 CI)	*p*	OR (%95 CI)	*p*
*Age*	0.978 (0.96–0.99)	**0.007**	0.97 (0.95–0.99)	**0.035**

*Gender*				
Female	1	—		
Male	0.97 (0.58–1.63)	0.932		

*TSH*	1.03 (0.96–1.12)	0.342		
*Hashimoto's*	0.98 (0.49–1.95)	0.962		

*MTN*				
No	1	—		
Yes	0.89 (0.58–1.36)	0.59		

*Nodule size*	0.99 (0.97–1.01)	0.69		
*Nodule number*	0.95 (0.88–1.03)	0.22		

*Ecogenicity*				
Isoechoic	1	—		
Hypoechoic	1.74 (1.12–2.70)	**0.013**		
Hyperechoic	0.97 (0.28–3.32)	0.973		
Mixed echoic	1.27 (0.51–3.13)	0.602		

*US findings*				
Cystic areas	1	—	1	—
Necrosis	6.46 (2.63–15.83)	**<0.001**	7.09 (2.65–18.96)	**<0.001**
Microcalcification	2.78 (1.45–5.32)	**0.002**	3.13 (1.53–6.41)	**0.002**
Macrocalcification	1.40 (0.30–6.48)	0.667	1.36 (0.27–6.86)	0.708
Border irregularity	13.44 (5.35–33.74)	**<0.001**	9.82 (3.37–28.63)	**<0.001**
Intermittent wall calcification	0.88 (0.11–7.05)	0.908	1.23 (0.14–10.35)	0.844
Taller than wide	38.40 (13.71–107.52)	**<0.001**	50.05 (14.67–170.73)	**<0.001**

*Doppler US*				
No blood flow	1	—	1	—
Peripheral blood flow	0.51 (0.26–1.02)	0.058	0.59 (0.25–1.37)	0.225
Central blood flow	3.55 (1.65–7.62)	**0.001**	2.88 (1.10–7.50)	**0.030**
Central and peripheral blood flow	1.58 (0.82–3.02)	0.167	1.46 (0.65–3.27)	0.357

*Sum of right and left lobes*				
Isthmus	1	—	1	—
Lower	4.45 (1.70–11.62)	**0.002**	8.39 (2.34–30.12)	**0.001**
Middle	2.24 (0.84–5.98)	0.105	4.27 (1.16–15.72)	**0.029**
Upper	4.57 (1.67–12.46)	**0.003**	8.09 (2.11–30.94)	**0.002**
Whole lobe	3.17 (1.12–8.96)	**0.029**	7.63 (1.95–29.81)	**0.003**

CI: confidence interval; OR: odds ratio.

## Data Availability

The data belong to the funders and are not available to the public in order to protect the patient privacy.

## References

[1] Singh Ospina N., Maraka S., Espinosa De Ycaza A. E. (2016). Physical exam in asymptomatic people drivers the detection of thyroid nodules undergoing ultrasound guided fine needle aspiration biopsy. *Endocrine*.

[2] Guth S., Theune U., Aberle J., Galach A., Bamberger C. M. (2009). Very high prevalence of thyroid nodules detected by high frequency (13 MHz) ultrasound examination. *European Journal of Clinical Investigation*.

[3] Tessler F. N., Middleton W. D., Grant E. G. (2017). ACR thyroid imaging, reporting and data System (TI-RADS): white paper of the ACR TI-RADS committee. *Journal of the American College of Radiology*.

[4] Russ G., Leboulleux S., Leenhardt L., Hegedüs L. (2014). Thyroid incidentalomas: epidemiology, risk stratification with ultrasound and workup. *European Thyroid Journal*.

[5] Nam I.-C., Choi H., Kim E.-S., Mo E.-Y., Park Y.-H., Sun D.-I. (2015). Characteristics of thyroid nodules causing globus symptoms. *European Archives of Oto-Rhino-Laryngology*.

[6] Kitahara C. M., Kӧrmendiné Farkas D., Jørgensen J. O. L., Cronin-Fenton D., Sørensen H. T. (2018). Benign thyroid diseases and risk of thyroid cancer: a nationwide cohort study. *The Journal of Clinical Endocrinology & Metabolism*.

[7] Sosa J. A., Hanna J. W., Robinson K. A., Lanman R. B. (2013). Increases in thyroid nodule fine-needle aspirations, operations, and diagnoses of thyroid cancer in the United States. *Surgery*.

[8] La Vecchia C., Malvezzi M., Bosetti C. (2015). Thyroid cancer mortality and incidence: a global overview. *International Journal of Cancer*.

[9] Cavallo A., Johnson D. N., White M. G. (2017). Thyroid nodule size at ultrasound as a predictor of malignancy and final pathologic size. *Thyroid*.

[10] Ito Y., Uruno T., Nakano K. (2003). An observation trial without surgical treatment in patients with papillary microcarcinoma of the thyroid. *Thyroid*.

[11] Meko J. B., Norton J. A. (1995). Large cystic/solid thyroid nodules: a potential false-negative fine-needle aspiration. *Surgery*.

[12] Haugen B. R., Alexander E. K., Bible K. C. (2016). 2015 American thyroid association management guidelines for adult patients with thyroid nodules and differentiated thyroid cancer: the American thyroid association guidelines task force on thyroid nodules and differentiated thyroid cancer. *Thyroid*.

[13] Russ G., Bonnema S. J., Erdogan M. F., Durante C., Ngu R., Leenhardt L. (2017). European Thyroid Association guidelines for ultrasound malignancy risk stratification of thyroid nodules in adults: the EU-TIRADS. *European Thyroid Journal*.

[14] Singh Ospina N., Brito J. P., Maraka S. (2016). Diagnostic accuracy of ultrasound-guided fine needle aspiration biopsy for thyroid malignancy: systematic review and meta-analysis. *Endocrine*.

[15] Shin J. H., Baek J. H., Chung J. (2016). Ultrasonography diagnosis and imaging-based management of thyroid nodules: revised Korean Society of Thyroid Radiology consensus statement and recommendations. *Korean Journal of Radiology*.

[16] Ramundo V., Lamartina L., Falcone R. (2019). Is thyroid nodule location associated with malignancy risk?. *Ultrasonography*.

[17] Jasim S., Baranski T. J., Teefey S. A., Middleton W. D. (2020). Investigating the effect of thyroid nodule location on the risk of thyroid cancer. *Thyroid*.

[18] Fukunari N., Nagahama M., Sugino K., Mimura T., Ito K., Ito K. (2004). Clinical evaluation of color Doppler imaging for the differential diagnosis of thyroid follicular lesions. *World Journal of Surgery*.

[19] Baskin H. J., Duick D. S., Levine R. A. (2013). *Thyroid Ultrasound and Ultrasound-Guided FNA Biopsy*.

[20] Lagalla R., Cariso G., Midiri M., Cardinale A. E. (1992). Echo-Doppler couleru et pathologie thyroidienne. *Journal of Echograph and Ultrasound in Medicine*.

[21] Baloch Z. W., LiVolsi V. A., Asa S. L. (2008). Diagnostic terminology and morphologic criteria for cytologic diagnosis of thyroid lesions: a synopsis of the national cancer institute thyroid fine-needle aspiration state of the science conference. *Diagnostic Cytopathology*.

[22] Evranos B., Polat S. B., Baser H. (2017). Bethesda classification is a valuable guide for fine needle aspiration reports and highly predictive especially for diagnosing aggressive variants of papillary thyroid carcinoma. *Cytopathology*.

[23] Hegedus L., Bonnema S. J., Bennedbæk F. N. (2003). Management of simple nodular goiter: current status and future perspectives. *Endocrine Reviews*.

[24] Reza R., Lisa Z., Electron K. (2010). Thyroid cancer gender disparity. *Future Oncology*.

[25] Zhang F., Oluwo O., Castillo F. B. (2019). Thyroid nodule location on ultrasonography as a predictor of malignancy. *Endocrine Practice*.

[26] Popowicz B., Klencki M., Lewiński A., Słowińska-Klencka D. (2009). The usefulness of sonographic features in selection of thyroid nodules for biopsy in relation to the nodule’s size. *European Journal of Endocrinology*.

[27] Gul K., Ersoy R., Dirikoc A. (2009). Ultrasonographic evaluation of thyroid nodules: comparison of ultrasonographic, cytological, and histopathological findings. *Endocrine*.

[28] Kumar H., Daykin J., Holder R., Watkinson J. C., Sheppard M. C., Franklyn J. A. (1999). Gender, clinical findings, and serum thyrotropin measurements in the prediction of thyroid neoplasia in 1005 patients presenting with thyroid enlargement and investigated by fine-needle aspiration cytology. *Thyroid*.

[29] Brito J. P., Gionfriddo M. R., Al Nofal A. (2014). The accuracy of thyroid nodule ultrasound to predict thyroid cancer: systematic review and meta-analysis. *The Journal of Clinical Endocrinology & Metabolism*.

[30] Moon H. J., Kwak J. Y., Kim M. J., Son E. J., Kim E.-K. (2010). Can vascularity at power Doppler US help predict thyroid malignancy?. *Radiology*.

[31] Chiaw L. C., Hong C. T., Chow W. T. (2018). Diagnostic performance of ATA, BTA and TIRADS sonographic patterns in the prediction of malignancy in histologically proven thyroid nodules. *Singapore Medical Journal*.

[32] Amisten S., Betts J. (2018). Can thyroid nodule localization predict thyroid cancer?. https://www.touchendocrinology.com.

[33] Sibai Z., Zahedi T. (2019). Thyroid nodule localization as a predictor factor for malignancy. *Journal of the Endocrine Society*.

[34] Pastorello R., Valerio E., Lobo A., Maia A., Saieg M. (2020). Do thyroid nodules that arise in the isthmus have a higher risk of malignancy?. *Cancer Cytopathology*.

